# Supplementary material for: Exploring regional disparities in lung cancer mortality in a Brazilian state: A cross-sectional ecological study
Source: PLoS One. 2023 Jun 23;18(6):e0287371. doi: 10.1371/journal.pone.0287371 (PMC10289318; doi:10.1371/journal.pone.0287371)
Supplement: Attachment — Submitted filename: Response to reviewers comments.docx [file pone.0287371.s001.docx]

Dear Fernanda Penido Matozinhos

Editor in chief of PloS One

We are sending the detailed response to the editor and reviewer’s comments to our manuscript entitled **“Exploring regional disparities in lung cancer mortality in a Brazilian state: a cross-sectional ecological study”.**

In the following table, we described how each editor and reviewer’s comment, sent in the revision of manuscript, was addressed.

| **Editor** | |
| --- | --- |
| **Editor’s comments** | **How each comment was addressed in the new revision of the manuscript** |
| 1. We note that Figures 1 to 3 in your submission contain [map/satellite] images which may be copyrighted. All  PLOS content is published under the Creative Commons Attribution License (CC BY 4.0), which means that the  manuscript, images, and Supporting Information files will be freely available online, and any third party is permitted to  access, download, copy, distribute, and use these materials in any way, even commercially, with proper attribution.  For these reasons, we cannot publish previously copyrighted maps or satellite images created using proprietary data,  such as Google software (Google Maps, Street View, and Earth). For more information, see our copyright guidelines:  http://journals.plos.org/plosone/s/licenses-and-copyright. | We would like to clarify that these figures were generated using the Brazilian shapefile that was obtained from the Brazilian Institute of Geography and Statistics (IBGE), freely available at: <https://www.ibge.gov.br/geociencias/organizacao-do-territorio/malhas-territoriais/15774-malhas.html?=&t=downloads>, in the archive “PR_municipios_2020.zip”.  In addition, in relation to Figure 1, part of it was generated using the South America shapefile, obtained freely at:  <https://tapiquen-sig.jimdo.com/english-version/free-downloads/south-america/>, and the open street map, obtained freely using a plugin “open street map” of software QGIS.  After obtaining these shapefile archives, the final maps with the spatial analysis results were produced using the QGIS software as described in the article´s Methods Section. These informations were further clarified in the article´s Methods Section and in the  legend of figures. |
| **Reviewer 1** | |
| **Reviewer’s comment** | **How each comment was addressed in the new revision of the manuscript** |
| 1. The paper explores the relationship between lung cancer mortality and some variables, regarding a Brazilian region. The procedures and techniques used seem sound and the conclusion follows from the results´ analysis. It is important to make a final revision of the text and to make the whole set of data available to the audience putting all of it in one place (in the web) | All the text was reviewed as requested and the whole set of data was made available to audience in the figshare website: <https://figshare.com/s/f6a8509b272a8c247a30> |
| **Reviewer 2** | |
| **Reviewer’s comment** | **How each comment was addressed in the new revision of the manuscript** |
| 1. The study presents an interesting spacial description and analysis of lung cancer cases in Parana. This kind of study is important to better destine public policies in the state. However, I miss some things in the discussion: what are the study strengths? And what are the health implications? What the managers can do with this information? For me, this study it's more important for this public, so they need to understand what is the scenario and what they can do. | We have reviewed and added this information more clearly in the discussion and conclusion sections as requested. |
| 2. The title is duplicated in the submission. | We excluded the duplicated title. |
| 3. Figures 2 and 3: The caption should be below the figure, not in the title. Figure 3 needs a title. The sentence there is the caption. | We are proceeding according to the journal's submission guidelines. Figure images are submitted separately from the main text of the manuscript. Figure legends are submitted within the text of the manuscript. The final format is presented according to the submission system settings. |
| 4. Line 302-304: Why these areas? Some people that will ready your work don't know Parana. I miss some explanation or hypothesis for this. It's because there are rural areas? Or because tobacco production occurs in these regions? | The discussion and conclusion sections were reviewed and more information was added to clarify our findings as requested. |

We look forward to further editorial orientations,

Best Regards

Vlaudimir Dias Marques

Corresponding Author
